# Tibial sagittal and rotational alignment reduce patellofemoral stresses in posterior stabilized total knee arthroplasty

**DOI:** 10.1038/s41598-022-15759-6

**Published:** 2022-07-19

**Authors:** Hideki Mizu-uchi, Yuan Ma, Shojiro Ishibashi, Clifford W. Colwell, Yasuharu Nakashima, Darryl D. D’Lima

**Affiliations:** 1 Department of Orthopaedic Surgery, Saiseikai Fukuoka General Hospital, 1-3-46, Tenjin, Chuo-ku, Fukuoka, 810-0001 Japan; 2 Department of Orthopaedic Surgery, Graduate School of Medical Sciences, Kyushu University, 3-1-1 Maidashi, Higashi-ku, Fukuoka, 812-8582 Japan; 3 Shiley Center for Orthopaedic Research and Education at Scripps Clinic, 10666 North Torrey Pines Road, MS126, La Jolla, CA 92037 USA

**Keywords:** Reconstruction, Preclinical research

## Abstract

Patellofemoral joint complications remain an important issue in total knee arthroplasty. We compared the patellofemoral contact status between cruciate-retaining and posterior-stabilized designs with varying degrees of tibial sagittal and rotational alignment using a computer simulation to ensure proper alignments in total knee arthroplasty. Knee kinematics, patellofemoral contact force and quadriceps force were computed using a musculoskeletal modeling program (LifeMOD/KneeSIM 2010; LifeModeler, Inc., San Clemente, California) during a weight-bearing deep knee bend. Two different posterior tibial slope (PTS)s (3° and 7°) and five different tibial tray rotational alignments (neutral, internal 5° and 10°, and external 5° and 10°) were simulated. Patellofemoral contact area and stresses were next computed using finite element analysis. The patellofemoral contact force for the posterior-stabilized design was substantially lower than the cruciate-retaining design after post-cam contact because of increasing femoral roll-back. Neutral rotational alignment of the tibial component resulted in smaller differences in patellofemoral contact stresses between cruciate-retaining and posterior-stabilized designs for PTSs of 3° or 7°. However, the patellar contact stresses in the cruciate-retaining design were greater than those in posterior-stabilized design at 120° of knee flexion with PTS of 3° combined with internal rotation of the tibial component. Our study provides biomechanical evidence implicating lower PTSs combined with internal malrotation of the tibial component and the resultant increase in patellofemoral stresses as a potential source of anterior knee pain in cruciate-retaining design.

## Introduction

Total knee arthroplasty (TKA) has become one of the most successful surgeries to relieve knee pain and restore knee function for the patients with osteoarthritis and rheumatoid knees. Although the reported survival rates of TKA are greater than 90–95% after 10–15 years^1,2^, patellofemoral (PF) joint complications remain an important issue because it can lead to component loosening, wear of polyethylene, joint instability, and revision of components^3–5^. In addition, poor clinical outcomes have been attributed to the PF joint, such as anterior knee pain, patella clunk syndrome, and patella subluxation^6–13^. Recent studies have suggested patella-component impingement as an additional source of polyethylene wear debris^13^.

PF contact is significantly affected by parameters such as patella thickness^14^, shape of patellar component^15^, and PF design features such as the shape and length of patellar groove^16^. Additionally, femorotibial kinematics can also affect PF biomechanics. A major difference between cruciate-retaining (CR) and posterior-stabilized (PS) designs is the existence of a post-cam, which is designed to substitute for the function of the resected posterior cruciate ligament (PCL) during knee flexion. The post-cam mechanism of the PS design more consistently induces femoral rollback, which should lower PF contact force. However, the relative effects of these CR versus PS design differences on PF contact have not been fully analyzed.

The alignment of the femoral and tibial components can also affect PF contact status by abnormally tilting or displacing the patella. However, achieving accurate postoperative alignment, particularly sagittal and rotational alignment of the tibial component, is difficult. Previous studies have found accurate tibial rotational alignment to be challenging and concluded that the percentage of cases with acceptable postoperative alignment is low (ranging from 38 to 46%)^17,18^. With respect to tibial sagittal alignment, Barrett et al. reported that in one-third of cases there was a difference of ± 2° between planned and actual posterior tibial slope (PTS)s even by high-volume surgeons^19^. It is therefore important quantify the effect of the tibial malalignments on PF contact status.

Most of the previous studies evaluating PF contact status were conducted in cadaveric knees^6,7,9,11,12,16^. However, it is technically challenging to apply large loads equivalent to multiples of body weights in cadaver experiments which tends to underestimate PF pressures^20^. In addition, surgical variations in bone cuts and inter-individual differences in bony anatomy and soft tissues tend to confound the analysis of PF contact. The effect of prosthesis design and postoperative alignment on PF contact conditions has yet to be fully established.

Computer models have been used to analyze the effect of prosthetic design, and component alignment on PF biomechanics^21,22^. We have also previously reported on computer models to predict postoperative knee kinematics after TKA^23–25^. Computer simulations can apply large loads and replicate weight-bearing activities using musculoskeletal models and quantify the effect of various factors while controlling for anatomy and soft tissue properties, which reduces the confounding effects of inter-individual differences^21^. In addition, computer simulation can be used to calculate quadriceps and patellar forces and PF contact status with high resolution, which cannot be measured in vivo^26^.

We used a computer simulation to compare the PF contact status between CR and PS designs with varying degrees of tibial sagittal and rotational alignment. The primary objective was to determine if PF contact stresses in the PS design would be lower than the CR design due to greater femoral rollback. The secondary objective was to determine the effect of PTS and axial malrotation of the tibial tray on PF contact status.

## Materials and methods

### Computer simulation

**Figure 1:**
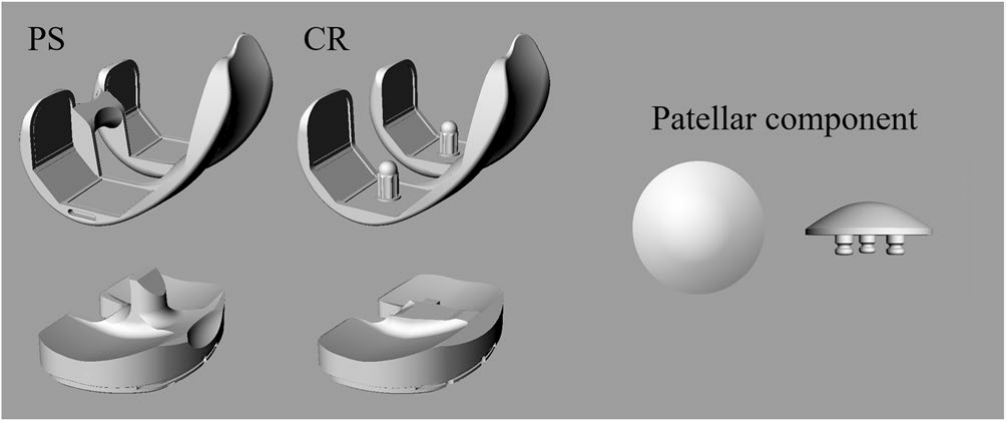
Cruciate-retaining and posterior-stabilized designs total knee system. *CR* Cruciate-retaining design, *PS* posterior-stabilized design.

All methods were carried out in accordance with relevant guidelines and regulations. We obtained approval from Institutional Review Board (Department of Orthopaedic Surgery, Graduate School of Medical Sciences, Kyushu University) and informed consent was obtained from the subject. We simulated weight-bearing deep knee flexion in a male patient (weight: 70 kg). The patient was implanted with a Scorpio PS total knee system (Stryker, MI, USA). For the simulation, we compared a CR design with a PS design (Fig. 1). The articular surfaces, except for the PS post-cam mechanism, were identical between these designs. Components sizes were selected to match the patient’s bony anatomy (femoral component: size 9, tibial component: size 9, insert: 10 mm, patellar component: 32 mm diameter and 8 mm thickness). Both femoral and tibial components were implanted perpendicular to the femoral and tibial mechanical axes in the coronal plane. The center of the patellar component was set at the center of the cutting surface of patella bone in a superior-inferior direction^26^. Implant components were aligned to the femur, tibia, and patella using a computer-assisted design (CAD) software program (Rhinoceros; Robert McNeel and Associates, Seattle, WA, USA). The origin of the coordinate systems for both CR and PS designs were defined as the centers of the inferior surface of the tibial insert, which was the intersection of the perpendicular bisector of the rectangle formed by the anterior–posterior and the medial–lateral dimensions. The most distal condylar points of the femoral component were aligned to the articular surface of the tibial insert in the superior–inferior direction.

**Figure 2:**
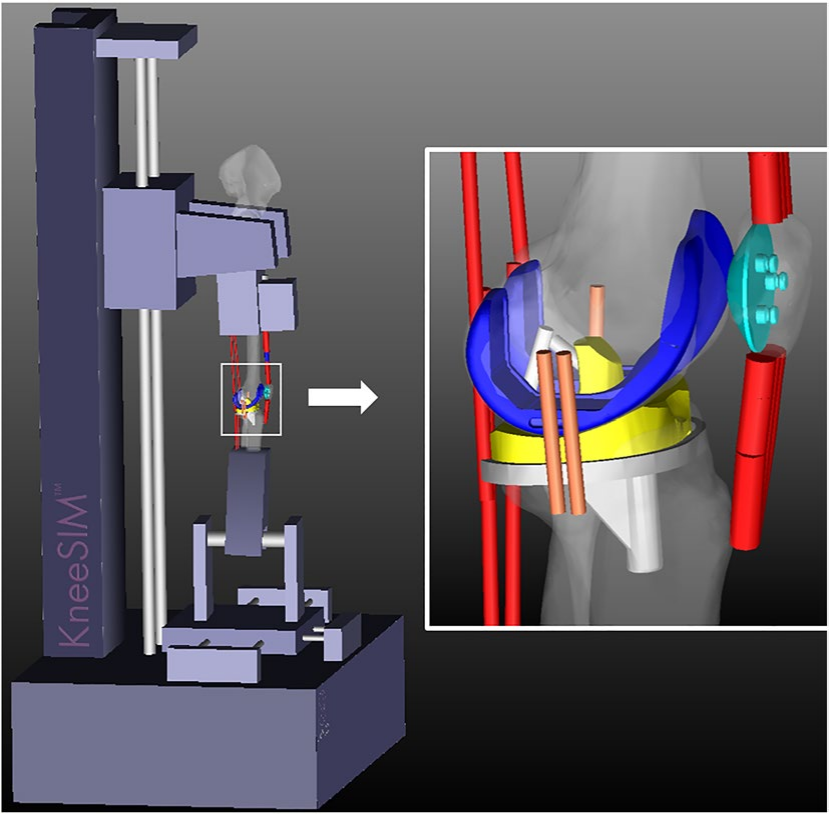
The knee simulator model used in the present study; LifeMOD/KneeSIM 2010.

The implant geometry was imported into a dynamic musculoskeletal modeling software (LifeMOD/KneeSIM 2010; LifeModeler, Inc., San Clemente, CA, USA; Fig. 2). KneeSIM uses rigid body dynamics to simulate a weight-bearing knee flexion similar to an Oxford type knee rig^23–29^. We have previously validated KneeSIM predicted kinematics and knee forces in cadavers and in patients^23–25,28,29^. The weight of the limb segments and upper body generate a flexion moment at the knee, whereas the quadriceps muscle exerts an extension moment. This musculoskeletal knee model included the quadriceps muscle and tendon, the hamstring muscles, the patellar tendon, the PCL (in the CR design), the medial and lateral collateral ligaments, patellofemoral ligaments, and patellotibial ligaments. Contact based wrapping was simulated between the quadriceps tendon and the femoral component was simulated as previously described^23,26,28^. The proximal attachment points of the bilateral collateral ligaments were aligned to the medial and lateral ends of the surgical epicondylar axis (SEA) respectively, which was also used to define the medial and lateral rotation centers of the femoral component. Collateral ligaments were modeled as nonlinear springs with previously published material properties^30^. Contact was simulated between the tibiofemoral and patellofemoral articular surfaces. The hip and ankle joints had three rotational degrees of freedom. The ankle section had no translational degrees of freedom. The hip section was constrained in the mediolateral and anteroposterior (AP) directions but was free to translate vertically in the direction of gravity.

### Evaluation of knee kinematics and forces

Knee kinematics, PF contact force and quadriceps force were computed from 0° to 120° of knee flexion. The AP translation of the femoral component and the locations of the lowest point of the medial and lateral femoral condyles (CLP) was tracked relative to the tibial component (anterior was positive and posterior was negative relative to the midline of the tibial insert). Kinematic measurements of the patellar component relative to the femoral component were analyzed during knee flexion. Patellar shift was measured as medial–lateral translation of the center of the component in the axial plane respective to the femoral component (medial shift: positive). The tilt of the patellar component was measured as the angle in the axial plane respective to the femoral component (lateral tilt: positive). We calculated Q angle as the abduction (lateral) angle between the patellar tendon and the quadriceps tendon over the range of flexion. Two different PTSs (3° and 7°) and five different tibial tray rotational alignments (neutral, internal 5° and 10°, and external 5° and 10°) were simulated in this study. The PTS was defined relative to the line connecting the center of the insert to the center of the ankle. Neutral tibial rotation was defined as parallel to femoral rotational alignment (both parallel to SEA).

### Evaluation of contact area and pressure on patellar component

Patellofemoral contact area and stresses were next computed using finite element analysis. CAD models of the patellar and femoral components were imported into Femap 12.0 with NX Nastran (Siemens PLM Software, Plano, TX, USA) and aligned based on the kinematic data output from the KneeSim model at knee flexion angles of 0°, 30°, 60°, 90° and 120°. The corresponding KneeSim computed PF contacting forces were applied to the components. The coefficient of friction was set to 0.04. The Young’s Modulus and Poisson’s Ratio were set as 220 GPa and 0.31 for the femoral components (Co–Cr–Mo alloy) and as 0.9 GPa and 0.45 for the patella component (ULMWPE). Both components were meshed with an average element size of 1 mm. The femoral components consisted of 276,904 nodes and 185,341 elements in CR design and 309,894 nodes and 207,659 elements in PS design. The patella components consisted of 32,679 nodes and 21,359 elements. The NX Nastran FEA solver was used to compute patellofemoral contact area and stresses. The PF contact area between patella component and femoral component was measured using Image-Pro Plus (Media Cybernetics, Inc., 401 N. Washington Street, Suite 350, Rockville, MD 20850, USA). The maximum von Mises equivalent stress of all the elements within 1.0 mm distance from the peaks of the stress distribution on the PF contact area was averaged to calculate peak stress.

### Ethical approval

All experiments were performed in accordance with relevant guidelines and regulations. Approved by Institutional Review Board (Department of Orthopaedic Surgery, Graduate School of Medical Sciences, Kyushu University: ID number of the approval 2019-432).

## Results

**Figure 3:**
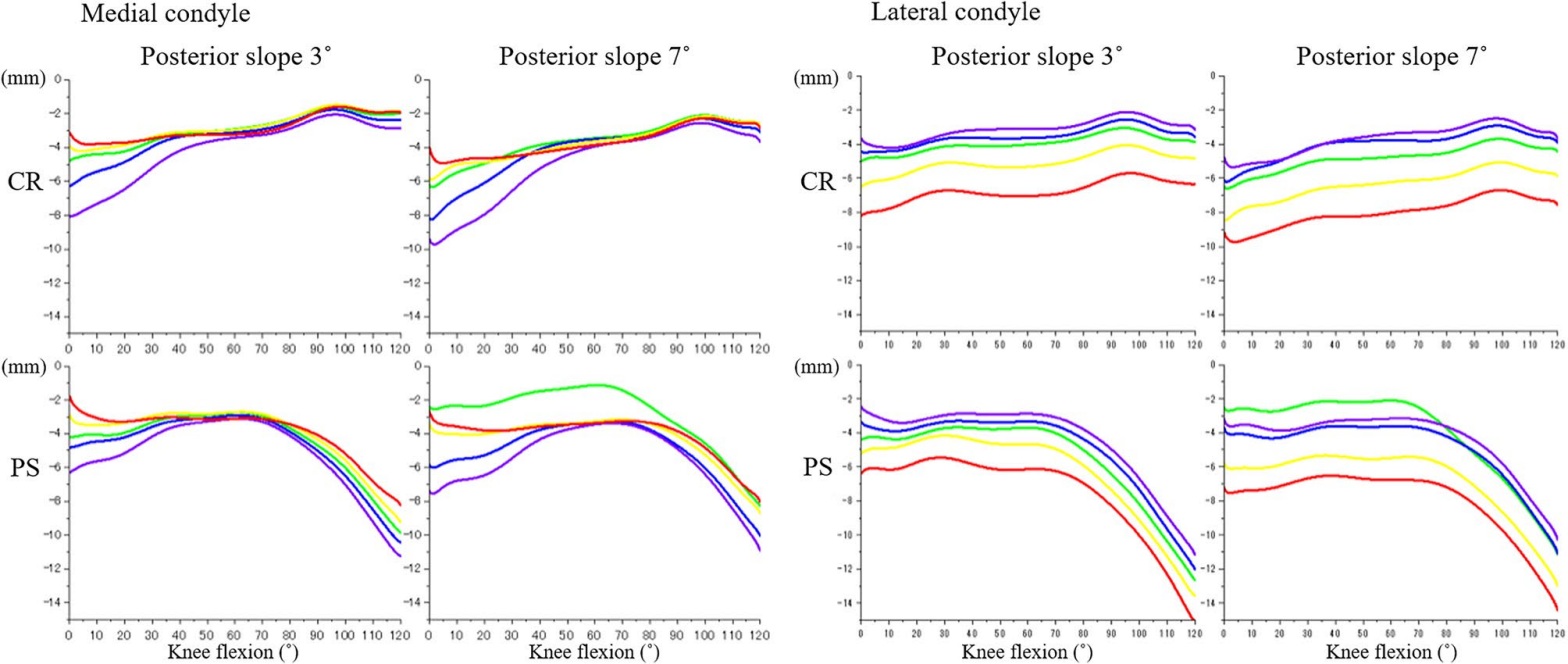
Condylar rollback of femoral component during knee flexion. *CR* cruciate-retaining design, *PS* posterior-stabilized design. Comparison between the tibial rotational alignments (red: internal 10°, yellow: internal 5°, green: neutral, blue: external 5°, purple: external 10°).

Overall, condylar rollback was markedly greater in PS relative to CR femoral components (Fig. 3). CR components did not exhibit much rollback, while PS components exhibited rollback after approximately 70° of flexion which corresponded with cam-post engagement. The lateral condylar rollback was greater than the medial condylar rollback and was reflected in the external rotation of the femoral component relative to the tibial component with knee flexion (Fig. 3). The PS design showed paradoxical femoral anterior movement in mid-flexion at 7° of PTS, not at 3° of PTS. Contact between the posterior aspect of the tibial post and the cam of the femoral component in the PS design was observed at around 75° of knee flexion (72° and 78° of knee flexion at 3° and 7° of PTS, respectively). From full extension to around 75° of knee flexion, the kinematics of the CR design were similar to that of the PS design. After post-cam contact, the PS femoral component translated posteriorly 9.5 mm more than the CR component, at 120° flexion. This was reflected in increased roll-back of the PS design when tracking the lowest points on the femoral condyles.

**Figure 4:**
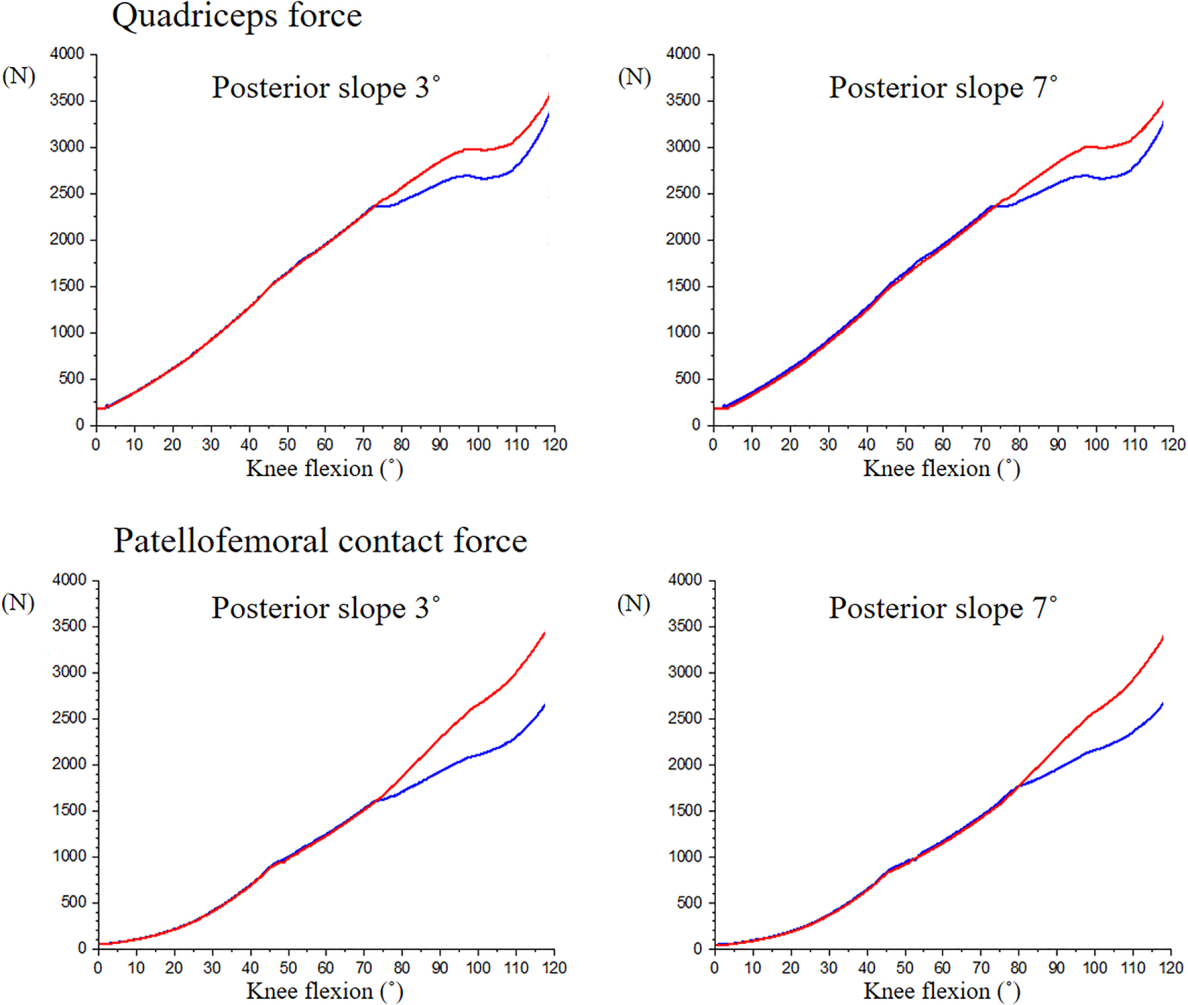
Quadriceps force and patellofemoral contact force of cruciate-retaining and posterior-stabilized design. Red: cruciate-retaining design, Blue: posterior-stabilized design.

**Figure 5:**
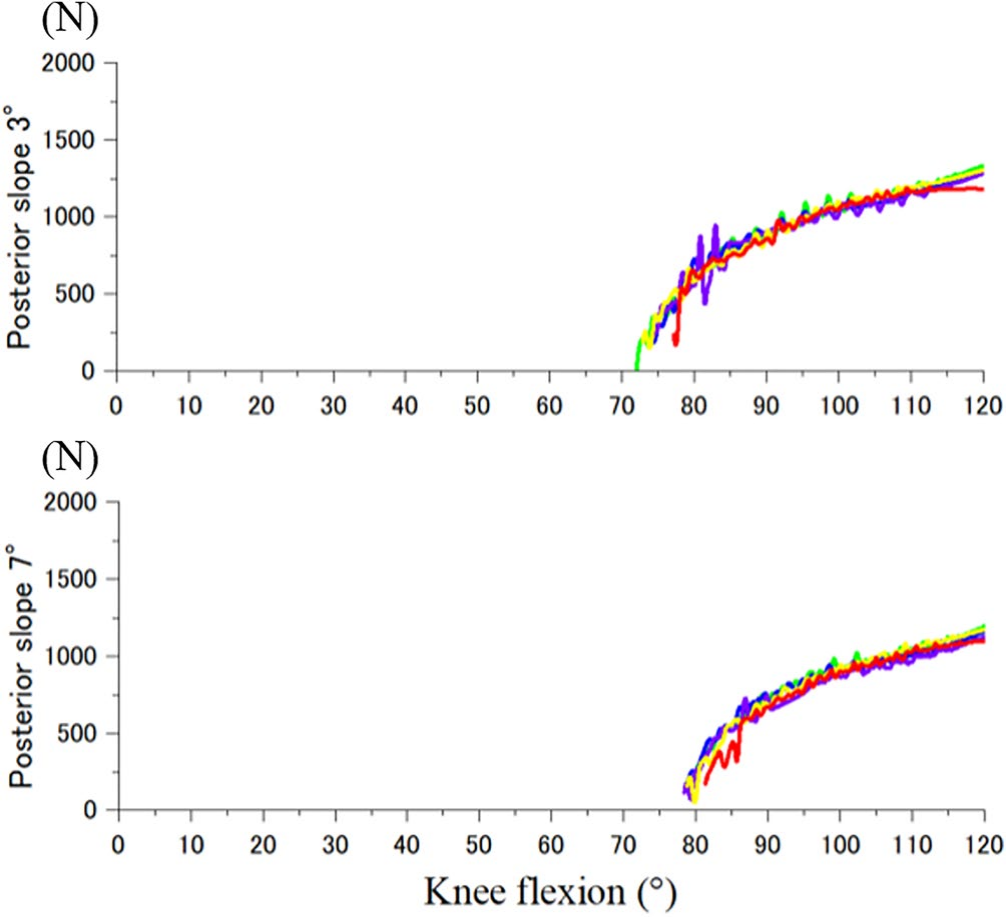
Posterior-cam contact force of posterior-stabilized design. Red: internal 10°, yellow: internal 5°, green: neutral, blue: external 5°, purple: external 10°.

**Figure 6:**
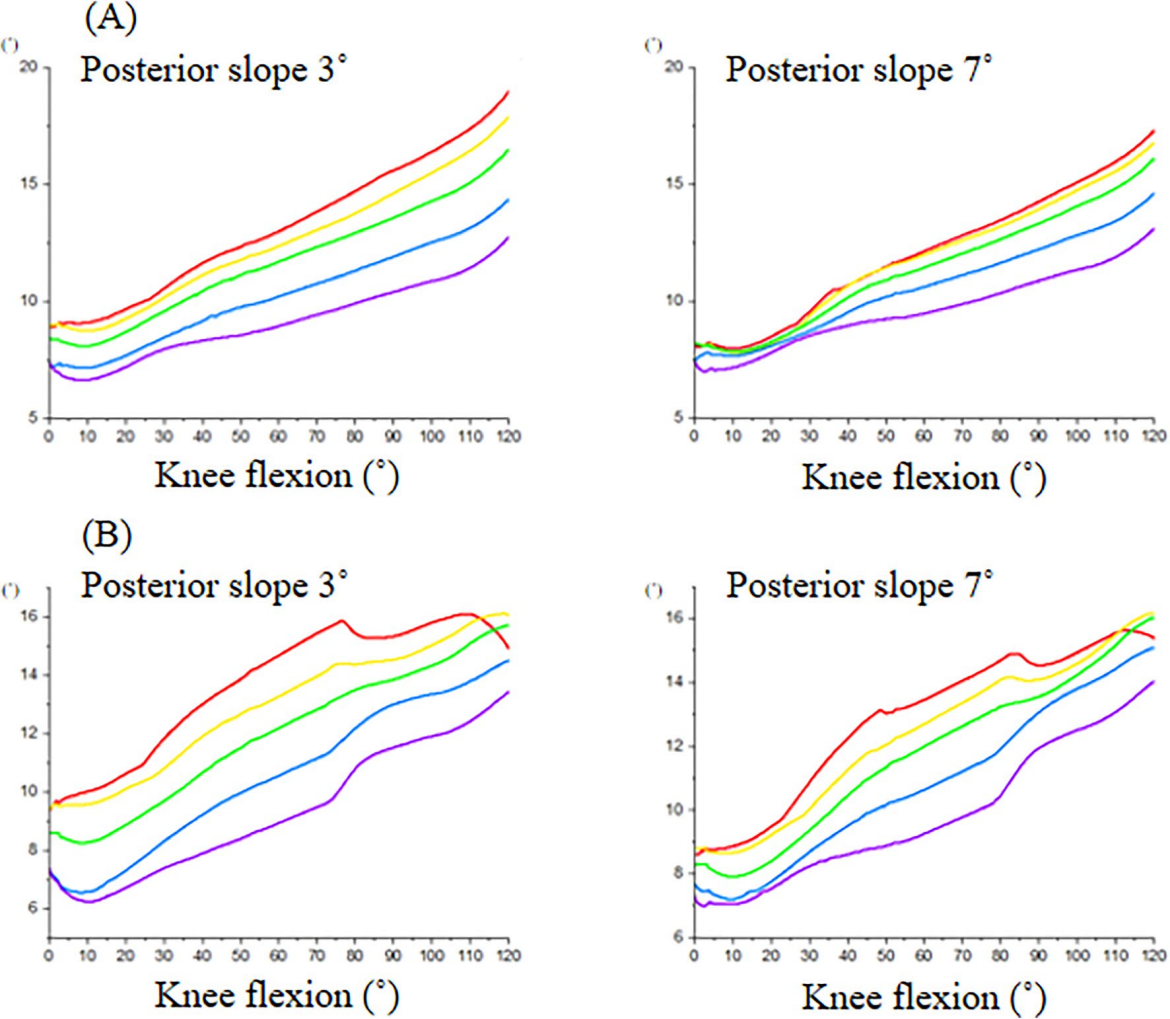
Q angle during knee flexion. (**A**) Cruciate-retaining design, (**B**) posterior-stabilized design. Comparison between the tibial rotational alignments (red: internal 10°, yellow: internal 5°, green: neutral, blue: external 5°, purple: external 10°).

**Figure 7:**
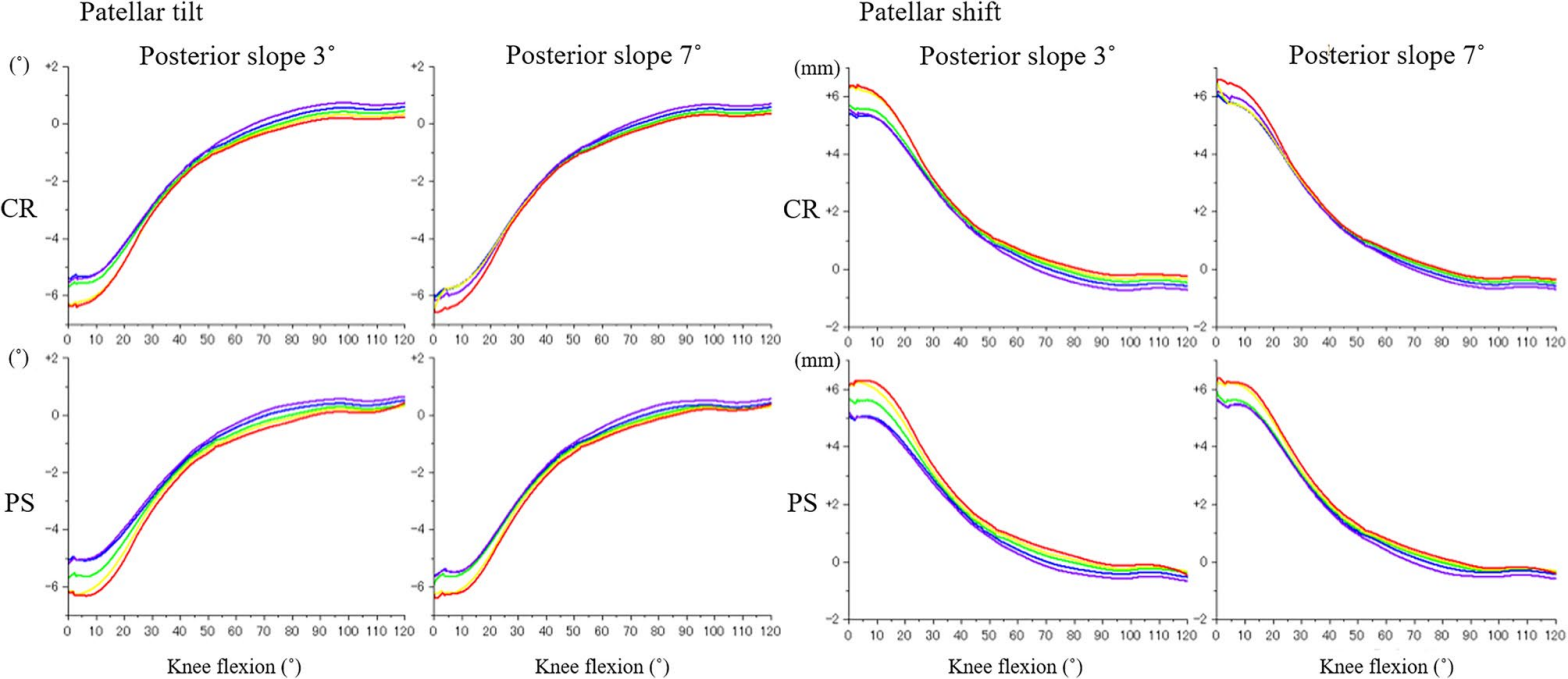
Patellofemoral kinematics during knee flexion. *CR* cruciate-retaining design, *PS* posterior-stabilized design. Comparison between the tibial rotational alignments (red: internal 10°, yellow: internal 5°, green: neutral, blue: external 5°, purple: external 10°).

Quadriceps force and PF contact force for CR and PS designs increased with knee flexion (Fig. 4). The forces for the PS design were similar to the CR design from extension to around 75° of knee flexion. After post-cam contact, PF contact force for the PS design was substantially lower than the CR design, reaching a difference of 1000 N at 120° knee flexion. The reduced PF contact forces for the PS design was associated with development of post-cam forces, after post-cam contact (Fig. 5). There were minimal differences in the patellar forces between 3° and 7° of PTSs among the rotational alignments of the tibial components. Internal rotation of the tibial component increased the Q angle of the extensor mechanism (Fig. 6). There were minimal changes in PF kinematics with implant design, PTS, or tibial component rotation (Fig. 7).

**Figure 8:**
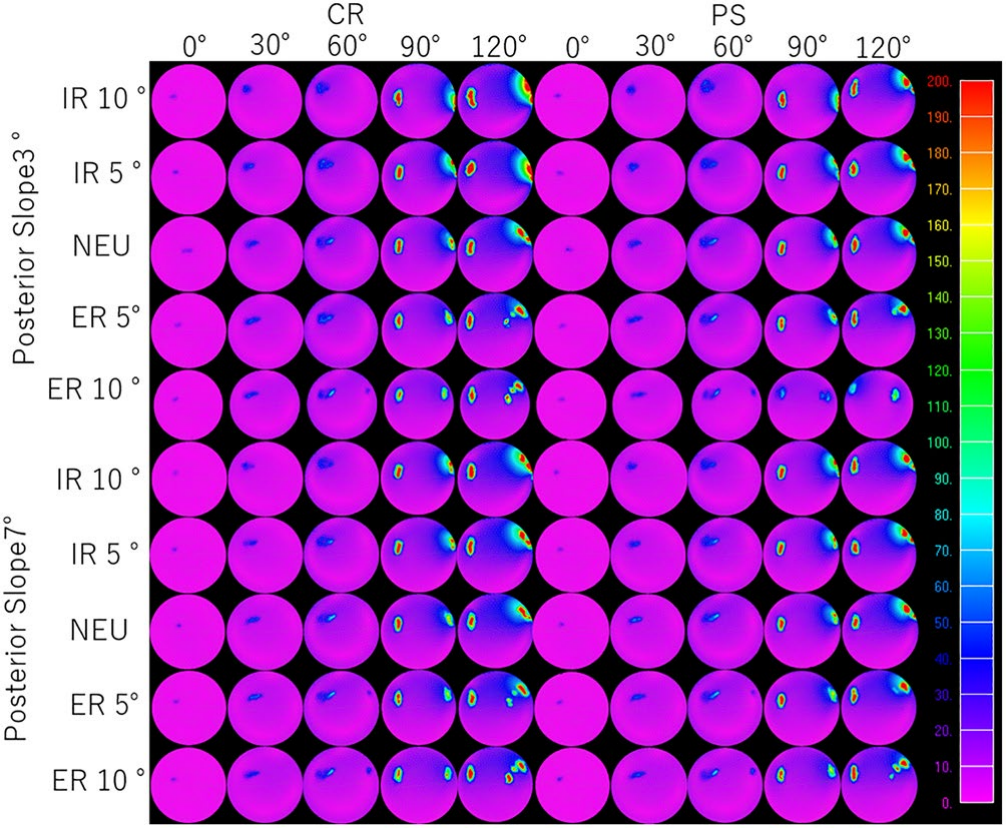
Distribution of von Mises equivalent stresses on the patellofemoral contact surface. *CR* cruciate-retaining design, *PS* posterior-stabilized design, *IR* internal, *NEU* neutral, *ER* external.

The contact area on the patellar component increased with knee flexion (Table 1). Figure 8 depicts medial and lateral stresses for the different implant conditions at various flexion angles. Internal rotation of tibial component resulted in smaller contact areas than neutral or external rotation. The medial contact area was concentrated near the medial edge of patella component surface of CR design under PTS of 3° at flexion of 90° and 120° (Fig. 8).

**Table 1:** Patellofemoral contact area during flexion.

			0°	30°	60° (l)	60° (m)	90° (l)	90° (m)	120° (l)	120° (m)
Posterior tibial slope of 3°	CR	IR 10°	5.81	15.91	25.15	NA	8.43	5.58	9.09	8.83
		IR 5°	7.37	20.41	29.17	NA	13.12	13.10	11.48	9.08
		NEU	7.02	17.90	28.32	NA	13.90	18.11	14.27	19.24
		ER 5°	6.46	21.62	27.62	NA	14.27	23.22	17.27	34.16
		ER 10°	7.22	20.57	27.62	6.00	17.00	19.10	17.84	33.90
	PS	IR 10°	6.60	12.49	25.10	NA	7.49	4.21	11.49	20.92
		IR 5°	7.44	15.83	27.84	NA	9.90	7.26	15.90	16.57
		NEU	10.13	18.21	23.15	NA	13.16	15.44	13.46	17.30
		ER 5°	6.77	22.01	28.05	NA	12.74	21.82	9.69	18.60
		ER 10°	5.94	24.63	28.25	9.25	22.11	25.80	14.87	21.48
Posterior tibial slope of 7°	CR	IR 10°	6.42	16.82	28.91	NA	11.27	15.75	11.43	21.58
		IR 5°	4.28	19.56	26.54	NA	10.85	16.41	16.70	22.48
		NEU	6.30	21.96	29.02	NA	12.66	19.00	16.50	22.02
		ER 5°	5.00	22.72	27.48	6.22	14.35	20.42	16.89	35.96
		ER 10°	6.04	16.77	28.65	6.27	17.95	18.60	16.01	34.79
	PS	IR 10°	6.79	13.81	28.10	NA	9.41	10.52	12.40	19.86
		IR 5°	5.55	20.77	29.41	NA	10.90	14.96	14.78	17.71
		NEU	6.17	19.26	28.13	NA	10.73	14.76	16.04	19.28
		ER 5°	5.94	23.06	26.99	5.44	12.62	17.39	13.03	23.16
		ER 10°	5.16	20.09	28.47	7.64	14.07	19.72	18.32	29.14

*CR* cruciate-retaining design, *PS* posterior-stabilized design, *IR* internal rotation, *NEU* neutral rotation, *ER* external rotation, *l* lateral, *m* medial, *NA* not available.

We analyzed the effect of implant design, knee flexion angle, and component orientation on PF stresses. The maximum von Mises equivalent stress on the patellar component increased with knee flexion (Fig. 8). Posterior tibial slope and tibial component rotation also affected the magnitude of maximum von Mises equivalent stress. In general, internal rotation of the tibial tray increased stresses, while increased PTS decreased contact stresses. Internal rotation of the tibial tray combined with a lower PTS generated the highest stresses. We noted a few differences between CR and PS designs. The patellar contact stresses in the CR design were greater than those in PS design at 120° of knee flexion with PTS of 3° with internal rotation of the tibial component. However, we found smaller differences in the contact stress of the patellar component between CR and PS designs with a PTS of 7°.

## Discussion

The most important findings of the present study were that PF contact forces in the PS design were lower than in the CR design after 75° flexion, and that contact stresses were higher in the CR design when the tibial component was internally rotated with a PTS of 3°. In contrast, when the tibial component was aligned in neutral axial rotation there were minimal differences in contact pressure between CR and PS designs. While in vivo studies are the most clinically relevant, the inherent variations in patient anatomy, kinematics, bone cuts, and implant position make controlled comparisons challenging^21^. Cadaver experiments can control external conditions, however, specimen-to-specimen differences in anatomy and soft tissues, and variation in surgical techniques can significantly affect knee kinematics and PF contact force^31^. Musculoskeletal computer models enable more controlled comparisons which facilitates quantification of the effect of individual parameters^21^.

Although PF contact forces increased with knee flexion, the PF contact force of the PS design was relatively lower than that of CR design beyond 75° of knee flexion. This was likely, due to greater rollback of femoral component induced by post-cam engagement at approximately 75°. A more posterior contact position between the femorotibial components increases the extensor moment arm, which improves the efficiency of the quadriceps resulting in reduced quadriceps force and therefore reduced PF contact force^32,33^. Defining optimal rollback is a multifactorial issue and varies with patient anatomy, implant design, and physical activity. Our results are consistent with Sharma’s analysis of PF contact forces based on in vivo fluoroscopy, which reported lower PF contact forces for the PS design compared to the CR design^34^. PF contact forces can be clinically relevant. Kaneko et al. found a negatively correlation between intraoperative compressive force across the PF joint at 140°of flexion and patient satisfaction and Forgotten Joint Score-12^10^.

PF stresses followed a trend similar to PF contact forces and peak von Mises stresses increased with knee flexion. Overall, there were minimal difference in PF stresses between CR and PS designs except when a PTS of 3° was combined with internal rotation of tibial component. The higher patellar stresses in the CR design at 3° could be related to the magnitude of femoral rollback. Increases in PTS contributed to a more posterior position of the femoral component (Fig. 3) which was associated with lower PF force (Fig. 4). Studies have found that greater PTS can protect the PCL by reducing posterior tibial translation in chronically injured PCL knees^35,36^. Our findings are also consistent with cadaver experiments that reported lower PF contact pressure in PS designs compared to CR designs^6,12^. However, these cadaver studies did not control for the effect of component malalignment on PF contact. On the other hand, Bauer et al. reported no differences in retropatellar contact pressure between PS and CR knees, but the patellae were not resurfaced, and external loads were low (50 N)^20^.

Tibial component malrotation in TKA is associated with pain, stiffness and abnormal PF kinematics^37–39^. Internal rotation of the tibial component results in a relative external rotation of the tibia which increases the Q angle and generates abnormal stresses on the patella and surrounding tissue. Nakagawa et al.^40^ showed that a 10° increase in the Q angle led to a 45% increase in maximal pressure under the patella. Kuriyama et al. reported that internal rotation of the tibial component induced internal rotation of the femoral component which in turn increased PF contact stresses^41^. Our finding that internal rotation of the tibial component was associated with high PF stresses and increased extensor Q angle is consistent with internal rotation of the femoral component. Excessive PF stresses could increase risk for anterior knee pain. Although computer assisted navigation and patient-specific instrumentation have increased the accuracy of coronal plane alignment, the error in axial rotation has not been reduced^18,42–44^. The percentage of cases with acceptable postoperative alignment is low^17,18^, and more than 90% of tibial trays were in internal rotation even after computer navigation^45^. Our results further emphasize the need for more accurate surgical techniques to avoid internal-malrotation of the tibial component to reduce complications due to increased PF pressure.

In contrast to coronal and rotational alignment, sagittal alignment, especially the acceptable range for PTS, remains controversial. Paradoxical femoral anterior movement in mid-flexion can be an issue with PS designs at greater PTS. In our study, we observed paradoxical femoral anterior movement at 7° of PTS, but not at 3° of PTS which is consistent with results reported by Okamoto et al.^25^. Lower PTSs with the CR design can cause tightness of the PCL^27,46^. Kuriyama et al. reported that 2° of increase in PTS, reduced PCL tension by over 40%^27^. In the CR design, we found lower rollback, and higher PF forces and stresses with 3° of PTS relative to the PS design, especially when combined with internal rotation of the tibial component. In contrast, PTS had minor effects on PF forces and stresses in the PS design.

Anterior knee pain is one of the more common postoperative complications after TKA. PF abnormalities can induce anterior knee pain, however, it is difficult to define the precise contribution of PF biomechanics because of the multifactorial nature of anterior knee pain. A few clinical studies have attempted to link PF biomechanics and clinical outcomes to CR and PS designs. Sharma^34^ used vivo fluoroscopic kinematics to compute PF forces and reported higher forces in the PS design relative to the CR design. Harato^8^ found the incidence of severe or moderate anterior knee pain to be greater in patients implanted with a CR design compared to a PS design in a prospective, randomized clinical trial. Our study provides biomechanical evidence implicating lower PTSs combined with internal malrotation of the tibial component and the resultant increased in PF stresses as a potential source of anterior knee pain in CR designs.

This study has several limitations. Only one prosthesis design with a single sagittal radius of curvature was used for the simulation. Other designs with different geometries and stability, such as medial pivot designs, can induce different kinematics. The Scorpio PS design we tested did not have an intercondylar box. Many PS designs have an intercondylar box which can reduce the length of the trochlear groove and consequently affect patellar stresses. We only studied one activity that simulated a deep knee bend. Other activities may generate different patellofemoral forces. The soft tissue material properties were based on the literature and were not subject-specific. However, the KneeSIM simulation software used in this study has been reported to be a useful tool and has been validated with cadaver and in vivo data^23–25,28,29^.

In summary, our computer simulation demonstrated lower PF forces and stresses with the PS design compared with the CR design, attributed to greater rollback. Neutral rotational alignment of the tibia component resulted in smaller differences in PF stresses between CR and PS designs for PTSs of 3° or 7°. However, a combination of PTS of 3° and mal-internal rotation of the tibial component generated the highest increase in contact stress between CR and PS designs.
